# Polyphosphate-Mediated Inhibition of Tartrate-Resistant Acid Phosphatase and Suppression of Bone Resorption of Osteoclasts

**DOI:** 10.1371/journal.pone.0078612

**Published:** 2013-11-04

**Authors:** Kana Harada, Hiromichi Itoh, Yumi Kawazoe, Shuichi Miyazaki, Kazuya Doi, Takayasu Kubo, Yasumasa Akagawa, Toshikazu Shiba

**Affiliations:** 1 Department of Advanced Prosthodontics, Institute of Biomedical & Health Sciences, Hiroshima University, Hiroshima, Japan; 2 Regenetiss Inc., Koganei, Tokyo, Japan; 3 Diagnostics Department, YAMASA Corporation, Choshi, Chiba, Japan; 4 The Kitasato Institute, Kitasato Institute for Life Sciences, Kitasato University, Tokyo, Japan; Faculté de médecine de Nantes, France

## Abstract

Inorganic polyphosphate (poly(P)) has recently been found to play an important role in bone formation. In this study, we found that tartrate-resistant acid phosphatase (TRAP), which is abundantly expressed in osteoclasts, has polyphosphatase activity that degrades poly(P) and yields Pi as well as shorter poly(P) chains. Since the TRAP protein that coprecipitated with anti-TRAP monoclonal antibodies exhibited both polyphosphatase and the original phosphatase activity, poly(P) degradation activity is dependent on TRAP and not on other contaminating enzymes. The ferrous chelator α, α’-bipyridyl, which inhibits the TRAP-mediated production of reactive oxygen species (ROS), had no effect on such poly(P) degradation, suggesting that the degradation is not dependent on ROS. In addition, shorter chain length poly(P) molecules were better substrates than longer chains for TRAP, and poly(P) inhibited the phosphatase activity of TRAP depending on its chain length. The IC50 of poly(P) against the original phosphatase activity of TRAP was 9.8 µM with an average chain length more than 300 phosphate residues, whereas the IC50 of poly(P) with a shorter average chain length of 15 phosphate residues was 8.3 mM. Finally, the pit formation activity of cultured rat osteoclasts differentiated by RANKL and M-CSF were markedly inhibited by poly(P), while no obvious decrease in cell number or differentiation efficiency was observed for poly(P). In particular, the inhibition of pit formation by long chain poly(P) with 300 phosphate residues was stronger than that of shorter chain poly(P). Thus, poly(P) may play an important regulatory role in osteoclastic bone resorption by inhibiting TRAP activity, which is dependent on its chain length.

## Introduction

Inorganic polyphosphate (poly(P)) is a polymer of tens to hundreds of orthophosphate (Pi) linked together by high energy phosphate bonds and is widely found in organisms ranging from bacteria to mammals [1]. In bacteria, various poly(P) functions, such as energy metabolism, survival, regulation of gene expression [2], translation fidelity [3], [4], motility, and virulence [5], [6] have been reported. In higher eukaryotes including mammals, several important poly(P) functions concerning bone regeneration [7], [8] and blood coagulation [9]–[12] have been recently described, suggesting that poly(P) also serves as a biologically active substance in mammals. In particular, stabilization of FGF by poly(P) during bone regeneration can positively regulate tissue regeneration, including bone formation [13], [14], and poly(P) induces the differentiation and calcification of osteoblasts [7], [15]. However, the detailed mechanisms underlying the effects of poly(P) on bone regeneration are largely unknown.

Tartrate-resistant acid phosphatase (TRAP; EC 3.1.3.2), which is also called type 5 acid phosphatase or purple acid phosphatase, is encoded by the *Acp5* gene in mammals and translated as a 35 kDa monomeric protein with low enzyme activity [16]. After translation, the monomer is proteolytically cleaved into two subunits, 22 kDa N-terminal and 16 kDa C-terminal fragments, which form an active heterodimeric enzyme through a disulphide bridge [17]. TRAP can dephosphorylate a number of substrates, including osteopontin, bone sialoprotein, casein, and mannose 6-phosphate [18], [19]. Moreover, TRAP is abundantly expressed on osteoclasts and plays an important role in osteoclastic bone resorption. For example, the resorbed bone matrix, such as type I collagen, is endocytosed into osteoclasts and is likely to be further degraded by reactive oxygen species (ROS) derived from TRAP [20]. Thus, the substrate specificity of TRAP is not high. In addition, TRAP seems to be secreted into the resorption lacuna and dephosphorylates bone matrix osteopontin, resulting in enhanced migration of osteoclasts [18], [21].

In this study, we found that TRAP has weak polyphosphatase.activity and that the phosphatase activity itself was inhibited by poly(P). Furthermore, we provide evidence showing that poly(P) inhibits the bone resorption activity of osteoclasts. Based on these findings, poly(P) could be a key molecule that regulates TRAP-mediated osteoclast bone resorption.

## Results

### rh-TRAP catalyzes the degradation of poly(P)

**Figure 1:**
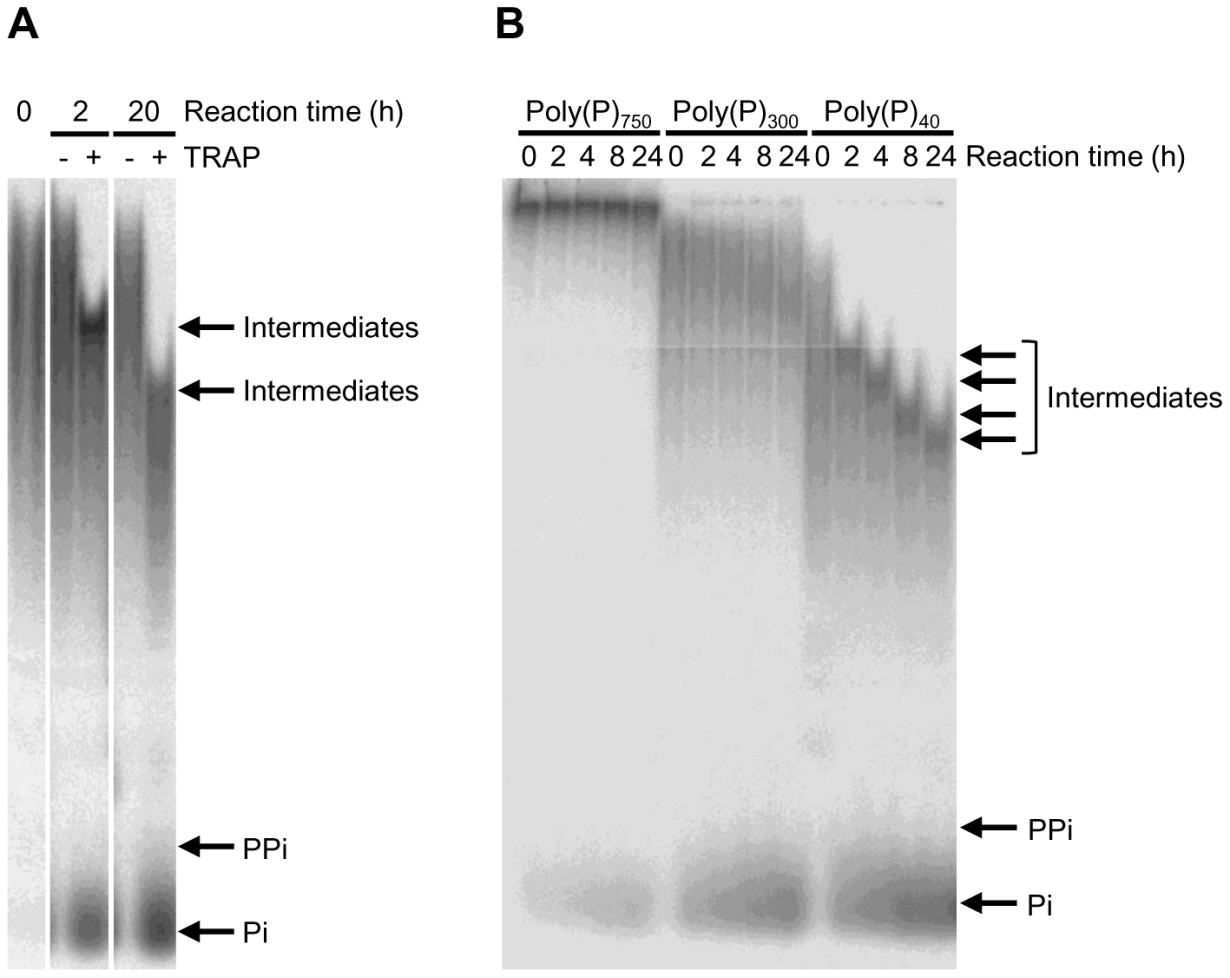
Degradation of poly(P) by Sf9 cell culture supernatant containing rh-TRAP. The [^32^P]-poly(P)_40_ (panel A) or [^32^P]-poly(P) with an average chain length of 40, 300, or 750 residues (panel B) (0.346 mM) were incubated with Sf9 cell culture supernatant containing rh-TRAP (7.3 mU/mL) in 100 mM Na-acetate buffer (pH 5.5) with 40 mM sodium tartrate for the indicated time periods at 37°C. Degradation products were analyzed by 20% PAGE.

We first examined whether the Sf9 cell culture supernatant containing rh-TRAP could degrade poly(P). As shown in Figure 1A, PAGE analysis revealed degradation of poly(P) having an average chain length of 40 phosphate residues (poly(P)_40_). Almost no degradation product was detectable when poly(P)_40_ was incubated in the reaction mixture without the culture supernatant. On the other hand, when the poly(P)_40_ was incubated with the culture supernatant, accumulation of Pi and intermediate poly(P) chains was detected. The length of the intermediate chain was shortened in a time-dependent manner.

We then examined the dependency of poly(P) degradation on the chain length. As shown in Figure 1B, poly(P) with an average chain length of 300 phosphate residues (poly(P)_300_) was also degraded by the culture supernatant, but the reaction speed was much slower than that of poly(P)_40_. When poly(P) had a longer average chain length of 750 phosphate residues (poly(P)_750_)_,_ very few degradation products were observed, including Pi. These results indicate that the culture supernatant containing rh-TRAP preferably degraded shorter chain length poly(P) and that the longer chain poly(P) is not suitable for the substrates. Moreover, we observed accumulation of intermediate poly(P) with a chain length of 20–60 phosphate residues.

To further confirm that such degradation of poly(P) is mediated by the rh-TRAP enzyme itself, we purified rh-TRAP from Sf9 cell culture supernatant by coprecipitation with anti-TRAP antibodies (clones 15A4 and 13B9). When rh-TRAP was coprecipitated with the anti-TRAP antibodies, polyphosphatase activity in the supernatants were the same level as the background control, respectively (Table 1). On the other hand, the pellet fractions from the coprecipitations with the two antibodies were capable of catalyzing poly(P)_40_ degradation at the same levels as the Sf9 cell culture supernatant (before coprecipitation). Measurement of the phosphatase activity using *p*-nitrophenylphosphate (*p*-NPP) as a substrate indicated that the pellet fractions exhibited approximately 90% of activity, while the supernatant fractions only had approximately 20% activity. Since the background level of phosphatase activity was 11.5%, less than 10% of the phosphatase activity remained in supernatant fractions. These results indicate that the rh-TRAP protein itself coprecipitating with anti-TRAP antibodies has phosphatase and polyphosphatase activity.

**Table 1:** Polyphosphatase activity as well as phosphatase activity of rh-TRAP after co-precipitation with anti-TRAP antibodies.

	Buffer	Before co-precipitation	Sup. (15A4)	Pellet (15A4)	Sup. (13B9)	Pellet (13B9)
Polyphosphatase	23.8±12.7	100.0*	22.7±18.3	103.3±6.08**	20.4±24.1	101.1±37.6***
Phosphatase	11.5±1.03	100.0^#^	21.7±0.80	88.7±2.95^##^	16.4±0.50	95.7±2.25^###^

The rh-TRAP was separated from Sf9 cell culture supernatant by co-precipitation with anti-TRAP antibodies (15A4 and 13B9). Polyphosphatase activity was also measured using [^32^P]poly(P)_40_ (0.346 mM) as substrate. Phosphatase activity was measured using *p*NPP as substrate. These activities were evaluated using the supernatant fraction (Sup.) or the pellet fractions (Pellet) after co-precipitation with anti-TRAP antibodies, 15A4 or 13B9. Values are expressed as means±SD of the percentage from three independent experiments comparing with the activities of Sf9 cell culture supernatant before co-precipitation as 100%. *p<0.05, significantly different from polyphosphatase activity of buffer. **p<0.05, significantly different from polyphosphatase activity of Sup. (15A4). ***p<0.05, significantly different from polyphosphatase activity of Sup. (15B9). ^#^ *p*<0.001, significantly different from phosphatase activity of buffer. ^##^ *p*<0.001, significantly different from phosphatase activity of Sup. (15A4). ^###^ *p*<0.001, significantly different from phosphatase activity of Sup. (13B9). (Student's t-test).

### Polyphosphatase activity of rh-TRAP is not dependent on ROS

**Figure 2:**
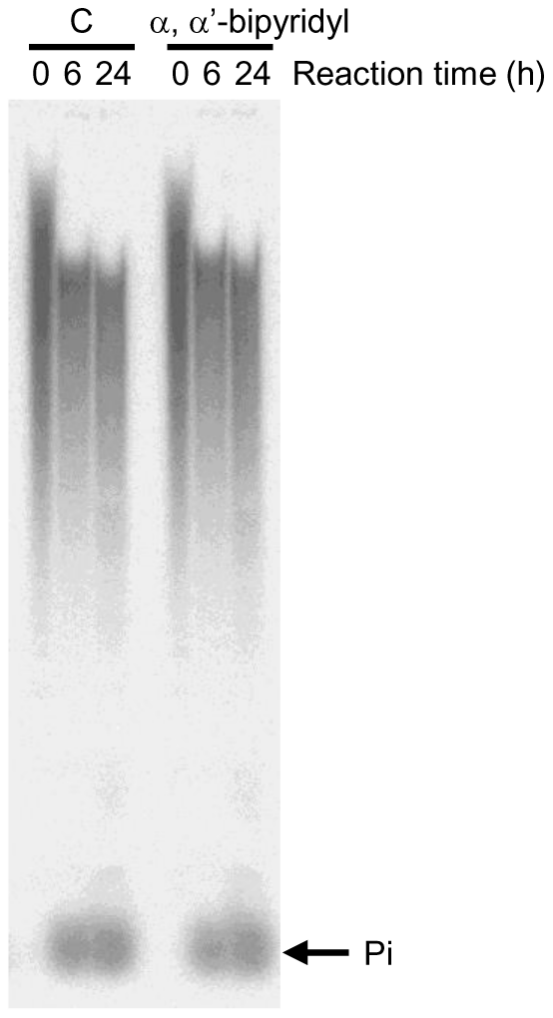
The effect of α, α’-bipyridyl, a ROS inhibitor, on polyphosphatase activity of rh-TRAP. The [^32^P]-poly(P)_40_ (0.346 mM) was incubated with recombinant human TRAP (7.3 mU/mL) in 100 mM Na-acetate buffer (pH 5.5) containing 40 mM sodium tartrate in the presence or absence of α, α’-bipyridyl (5 mM) for the indicated time periods at 37°C. The degradation of [^32^P]-poly(P) was analyzed by 20% PAGE.

The catalytic center of TRAP contains a redox-active iron, which can generate ROS through the Fenton reaction [22]. Thus, we examined the possibility that ROS was involved in the poly(P) degradation by TRAP. Alpha, α’-bipyridyl, which is a ferrous chelator, inhibits TRAP-mediated ROS production without affecting the phosphatase activity [23]. Treatment of TRAP with 5 mM α, α’-bipyridyl, which is a sufficient concentration for inhibiting ROS [23], did not affect the degradation of poly(P) or the generation of Pi (Figure 2), suggesting that the polyphosphatase activity of TRAP was not due to ROS production.

### Longer chain poly(P) is a potent inhibitor of rh-TRAP phosphatase activity

Since rh-TRAP has both phosphatase and polyphosphatase activities, we examined whether the poly(P) could be an inhibitor of the rh-TRAP phosphatase activity. TRAP substrates can inhibit the dephosphorylation of other substrates due to the low substrate specificity of TRAP [16]. Table 2 shows IC_50_ values for each length of poly(P) chains toward TRAP-catalyzed dephosphorylation of *p*NPP. Long chain poly(P) (poly(P)_300_ and poly(P)_750_) strongly inhibited the dephosphorylation of *p*NPP, which appeared to be dependent on the poly(P) chain length.

**Table 2:** IC_50_ values of each chain-length of poly(P) for rh-TRAP activity.

Poly(P)	IC_50_±SD (μM)
Poly(P)_750_	0.663±0.0242
Poly(P)_300_	4.43±0.395
Poly(P)_40_	47.6±2.27
Poly(P)_15_	846±86.7

*p*NPP (8 mM) were incubated with rh-TRAP in the reaction mixture in the presence or absence of the indicated chain-length of poly(P) (10^−8^, 10^−7^, 10^−6^, 10^−5^, 10^−4^, 10^−3^ or 10^−2^ M) for 2 h at 37°C. IC_50_ was average value of three independent experiments and determined by plotting the inhibition rate of phosphatase activity versus the logarithm of poly(P) concentration. SD represents standard deviation.

### Poly(P) suppresses the bone resorption activity of osteoclasts

**Figure 3:**
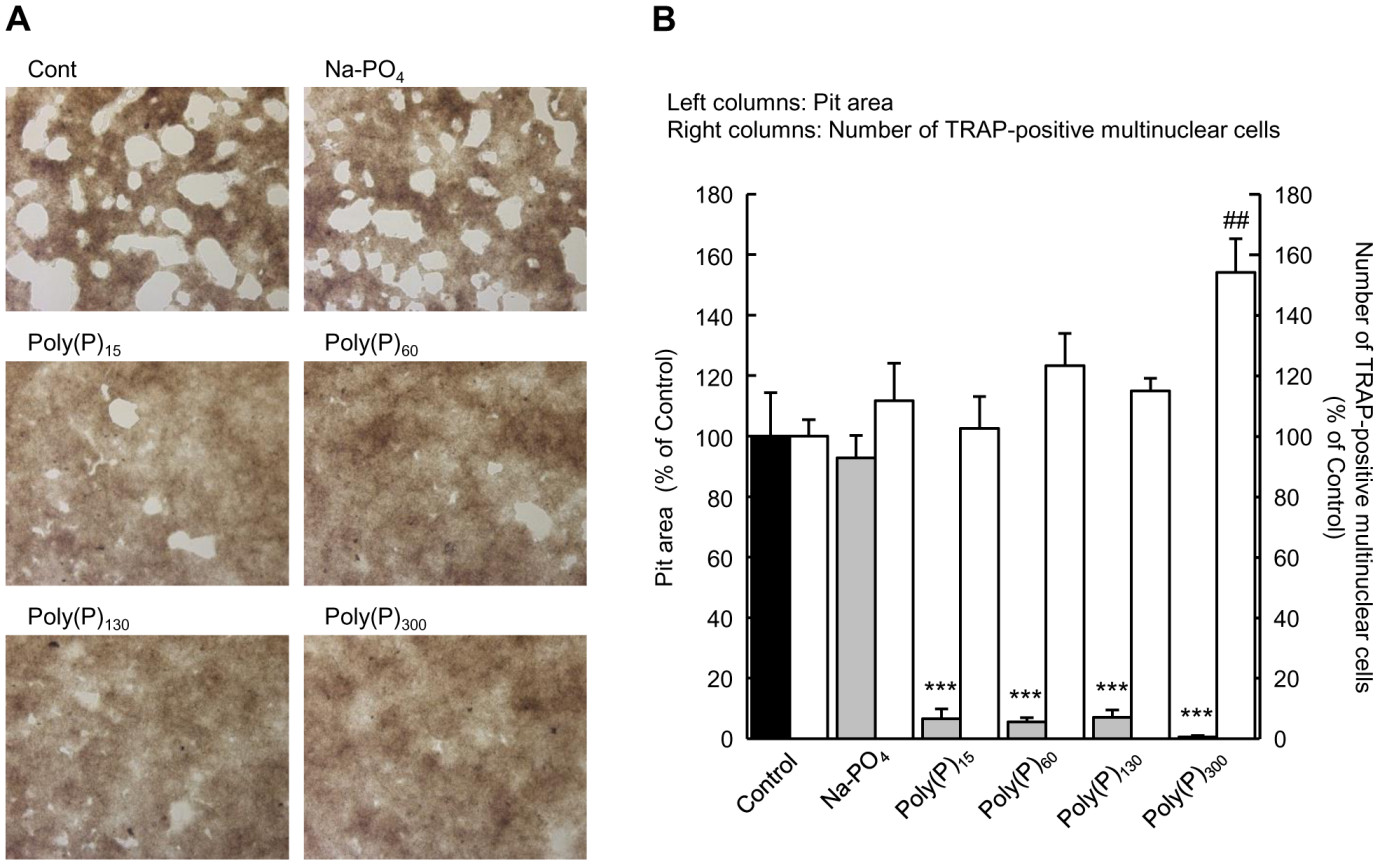
Inhibition of osteoclastic resorption activity by poly(P). The osteoclast precursor cells were plated in calcium phosphate-coated plates (Bone Resorption Assay Plate 24) and stimulated with M-CSF and RANKL. After 3 days in culture, the cells were treated with the indicated lengths of poly(P) (1 mM) and incubated for an additional 2 days (for TRAP staining) or 4 days (for resorption activity assay). (A) Images of pits obtained by bright field microscopy. (B) The pit (white) area measured by image analysis. Total white areas visualized by bright microscopy were summed using image analysis software (Image J). The number of TRAP-positive multinuclear cells was counted after the cells were stained for TRAP activity. Values are expressed as means±SD. ***p<0.001, significantly different from the pit area of control. ^##^ *p*<0.01, significantly different from the number of TRAP-positive cells of control (ANOVA with Bonferroni's post-test).

Since TRAP is involved in bone resorption by osteoclasts [24]–[27], we examined whether poly(P) could influence the bone resorption activity of osteoclasts. When osteoclast precursor cells obtained from bone marrow were stimulated with M-CSF and RANKL, multinuclear cells, which are differentiated osteoclasts, appeared after 3 days of culture and formed a number of pits in the calcium phosphate layer within 7 days of culture (Figure 3A). When 1 mM poly(P) was added at day 3 of the culture, the number of pits present was remarkably decreased by day 7 (Figures 3A and 3B). In particular, longer chain poly(P) (poly(P)_300_) almost completely inhibited pit formation. Since the number of TRAP-positive multinuclear cells did not decrease after the addition of poly(P) into the culture media, we concluded that poly(P) do not inhibit osteoclast differentiation (Figure 3B). In the opposite manner, poly(P)_300_ significantly increased the number of TRAP-positive multinuclear cells.

## Discussion

Although many polyphosphatases have been identified in microorganisms to date, little is known regarding these enzymes in mammals. Polyphosphatases are classified into exopolyphosphatases and endopolyphosphatases according to the manner of poly(P) degradation. Exopolyphosphatases hydrolyze the terminal phosphate bonds of poly(P) to release Pi, while endopolyphosphatases cleave the internal phosphate bonds and generate intermediate poly(P) chains. Lorenz et al. first identified exopolyphosphatase activity in mammalian cells [28], and Kumble and Kornberg identified endopolyphosphatase in *S. cerevisiae* and mammals [29]. Lorenz et al. also found that mammalian alkaline phosphatase can hydrolyze poly(P) to Pi [30]. More recently, Tammenkoski et al. showed that the human metastasis regulator protein H-Prune is a short-chain exopolyphosphatase [31].

In the case of TRAP, the degradation of poly(P) resulted in the generation of Pi, indicating that TRAP also has exopolyphosphatase activity. Gel analysis showed that poly(P) with a shorter chain is a more preferable substrate for TRAP than poly(P) with a long chain. Since the longer chain poly(P) has lower concentrations of terminal end in its molecule, TRAP may not be able to attack efficiently in the longer chain. On the other hand, shorter chain poly(P) has higher concentrations of terminal end than that of longer chain. Thus, shorter chain poly(P) might be more preferable substrate for TRAP. In addition, intermediate poly(P) chains with approximately 20 to 60 phosphate residues accumulated during the degradation reaction and gradually shortened in a time-dependent manner. (Figures 1A and 1B). These results show that the degradation reaction is not processive and that such intermediate chains may form relatively stable enzyme-substrate complexes with TRAP. Since the phosphatase activity of TRAP was inhibited by poly(P) in a chain length-dependent manner, longer chain poly(P) could bind to TRAP with higher affinity than shorter chains and efficiently inhibit enzyme activities. Although poly(P) with shorter chains can also bind the enzyme, it may still be degraded by TRAP and may not continuously inhibit enzyme activity.

Ruiz et al. and Müller et al. previously reported that the average chain length of intracellular poly(P) in the granules of human platelets is estimated to be 60–100 phosphate residues [11], [32]. Moreno-Sanchez et al. also showed that poly(P) in RBL-2H3, which is rat basophilic leukemia cell line, has approximately 60 phosphate residues [33]. These findings support the hypothesis that TRAP may contribute to the production of poly(P) with 60 phosphate residues, which could be functional in mammals. If longer poly(P) chains are synthesized, then they would be gradually degraded by TRAP and accumulate as intermediate chains.

We found that the rh-TRAP that coprecipitated with two different anti-TRAP antibodies had both phosphatase and polyphosphatase activity, which confirmed that the phosphatase activity was a result of the protein itself and not derived from other contaminating enzymes (Table 1). Furthermore, the degradation of poly(P) by TRAP was not due to ROS generation, suggesting that the degradation activity is not a non-specific reaction (Figure 2).

TRAP is encoded by a single gene, *Acp5* [34]. *Acp5*-deficient mice were reported to show mild osteopetrosis [26]. In addition, treatment of osteoclasts with TRAP inhibitors leads to the reduced bone resorption [24], [25]. Similarly, the poly(P)-mediated inhibition of the phosphatase activity of TRAP could be attributed to the suppression of calcium phosphate resorption.

Recent studies have shown that poly(P) in platelets plays an important role in blood coagulation. Platelet poly(P) is stored in the granules [32] and released in response to platelet activators, such as ADP and thrombin [11]. Therefore, poly(P) granules that form from intermediate chains may be physiologically important. Omelon et al. are suggested the existence of granules containing poly(P) in osteoclasts [35]. Schröder et al. also reported that a relatively high concentration of poly(P) is found in osteoblasts [36]. In our previous studies, we showed that poly(P) promotes osteoblast differentiation and calcification [7], [15]. In addition, poly(P) significantly inhibited osteoclastic resorption of calcium phosphate in this study. These findings suggest that poly(P) plays an important role in the regulation of bone regeneration. Poly(P) promotes bone formation by accelerating osteoblast differentiation and suppressing osteoclastic bone resorption. Since poly(P) itself efficiently traps Ca^2+^, it may locally concentrate Ca^2+^ and create a preferable environment for bone formation. On the other hand, osteoclasts also degrade poly(P) and promote favorable conditions for bone resorption. Also, poly(P)_300_ significantly enhanced cell division of precursor cells or differentiation of TRAP-positive multinuclear cells. Since poly(P) stabilize FGF-1 and FGF-2 and enhance binding affinity between FGF-2 and its cell surface receptors [13], poly(P) may also stabilize and enhance binding affinity of growth factors and/or differentiation factors such as M-CSF and RANKL.

The chain length of poly(P) may also be important for its regulatory role in bone regeneration. Poly(P) with more than 15 phosphate residues inhibits bone resorption (Figure 3), and poly(P) with a longer chain is a better inhibitor of TRAP (Table 2), suggesting that poly(P) with a relatively longer chain could be a regulatory factor for bone regeneration. Since certain chain lengths of poly(P) accumulated as intermediates in the TRAP enzymatic reaction, these intermediates (approximately 20–60 phosphate residues) may also be key molecules for bone regeneration.

**Figure 4:**
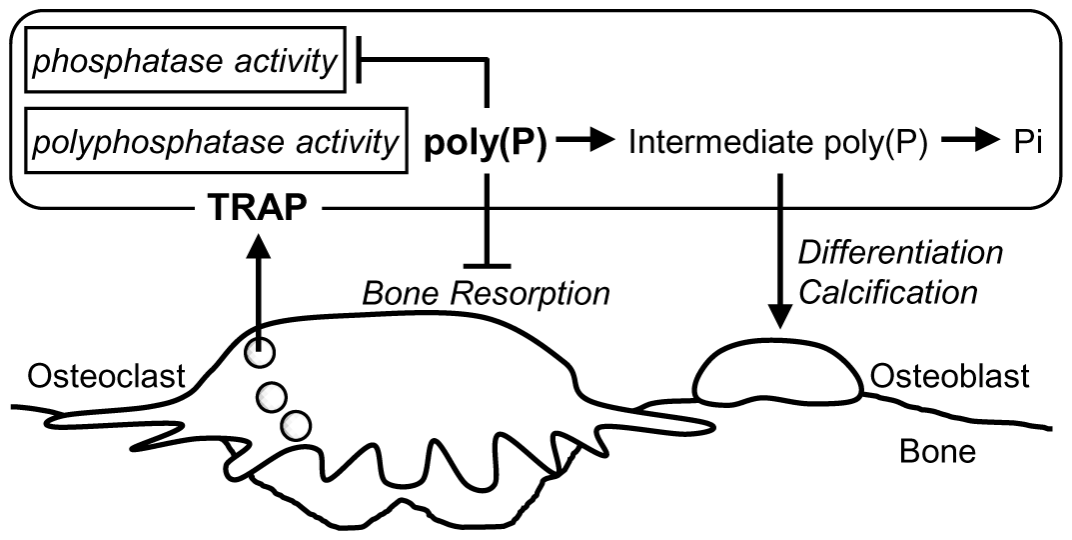
A schematic model of poly(P) function in bone remodeling. TRAP, which is secreted from osteoclasts, degrades poly(P) into intermediate poly(P) and subsequently into Pi. Poly(P) inhibits osteoclastic bone resorption, while intermediate poly(P) promotes differentiation and calcification of osteoblasts [7], [15]. Thus, poly(P) may regulate bone remodeling.

Based on the findings of this study, we propose a probable model of poly(P) function in bone remodeling (Figure 4). When bone resorption is activated, the TRAP enzyme in lysosomes of osteoclasts is secreted from the basolateral surface into the extracellular space [20] as well as from the ruffled border into the resorption lacuna [37]. Since poly(P), but not Pi, suppresses the osteoclastic resorption activity, degradation of poly(P) by the secreted TRAP could weaken and finally extinguish the poly(P)-mediated suppression of bone resorptioin. According to the hypothesis presented by Omeron et al., poly(P) may be utilized by osteoblast to construct new bone [35]. This proposal is consistent with our previous findings that poly(P) induces osteoblast differentiation and calcification [7], [15]. These data support the hypothesis that intermediate chains of poly(P) that consist of approximately 20–60 phosphate residues produced by TRAP may induce bone formation by osteoblasts.

The detailed mechanisms underlying poly(P)-mediated inhibition of osteoclastic resorption of calcium phosphate remains to be elucidated. However, involvement of poly(P) in bone remodeling could be a key point in the development of novel therapeutic strategies for bone diseases.

## Materials and Methods

### Materials

The Osteoclast Culture Kit (rat) and TRAP staining kit were obtained from Primary Cell Co., Ltd. (Hokkaido, Japan). The Bone Resorption Assay Plate 24 was obtained from PG Research (Tokyo, Japan). Poly(P) was prepared and its average chain length was determined by gel electrophoresis as previously described [7]. [^32^P]-poly(P) was enzymatically synthesized using purified *E. coli* polyphosphate kinase together with [γ-^32^P]-ATP, and [^32^P]-labeled poly(P)_750,_ poly(P)_300_, and poly(P)_40_ were prepared as previously described [4]. The pH of each poly(P) stock solution was adjusted to 6.8 – 7.0. The [^32^P]-pyrophosphate and [^32^P]-Pi were generated by luciferase-catalyzed hydrolysis of [γ-^32^P]-ATP and rPPX1-catalyzed hydrolysis of [^32^P]-poly(P), respectively [38]. Recombinant human TRAP (rh-TRAP) and anti-TRAP monoclonal antibodies (clones 15A4 and 13B9) were prepared as previously described [38]. Sf9 cells were purchased from Life Technologies Corporation (Carlsbad, US). All other reagents were purchased from commercial sources and were of the highest available purity.

### Measurement of polyphosphatase activity

The [^32^P]-poly(P) was incubated with rh-TRAP (7.3 mU/mL) in 100 mM Na-acetate buffer (pH 5.5) containing 40 mM sodium tartrate at 37°C. After incubation, the reaction products were separated by 20% polyacrylamide gel electrophoresis (PAGE) in 40 mM Tris-Acetate (pH 8.3) and 1 mM EDTA. Alternatively, the reaction products were applied to polyethyleneimine-thin layer chromatography (PEI-TLC) plates and developed with 2 M LiCl and 1 M HCOOH. Reaction products were visualized by exposing the gel or the TLC plates to an imaging plate and analyzed by FLA3000 (Fuji film, Tokyo). All of the poly(P) concentrations were presented in terms of phosphate residues. The ferrous chelator, α, α’-bipyridil, was also added to the reaction mixture at a final concentration of 5 mM if necessary [23].

### Identification of polyphosphatase activity using anti-TRAP monoclonal antibody

Culture supernatant of Sf9 cells expressing rh-TRAP was prepared as previously described [39]. The supernatant was incubated with the anti-TRAP monoclonal antibodies (15A4 or 13B9) separately in 0.2 M Na-acetate buffer (pH 5.5) for 10 min at 4°C. Protein G Sepharose beads were then added to the mixture and incubated for 10 min. After centrifugation, the pellet and supernatant fractions were separated. The rh-TRAP-mediated phosphatase and polyphosphatase activities both in the pellet and the supernatant fractions were evaluated.

### Measurement of rh-TRAP-mediated phosphatase activity

The rh-TRAP-mediated phosphatase activity was measured using *p*NPP (8 mM) as substrate in 50 µl of 100 mM Na-acetate buffer (pH 5.5) containing 40 mM sodium tartrate at 37°C. After incubation, 50 µl of 0.05 N NaOH was added to the reaction mixture to convert *p*-nitrophenol to *p*-nitrophenolate. To quantify the resulting reaction product, *p*-NPP, the absorbance at a 405 nm wavelength was measured.

### Preparation and identification of osteoclasts

The osteoclast precursor cells prepared from rat bone marrow were purchased from Primary Cells Co., Ltd., and cultured in α-MEM supplemented with 10% fetal bovine serum, 50 ng/mL M-CSF, and 15 ng/mL RANKL at 37°C with 5% CO_2_. To differentiate the precursor cells into osteoclasts, cells were incubated for 3 days and further incubated for 4 days with or without poly(P). To identify osteoclasts, the cells were stained for TRAP activity. The cells were washed with phosphate buffered saline (PBS) and fixed with 10% neutral buffered formalin for 5 min at room temperature. After washing with deionized water, the cells were incubated with chromogenic substrate in Na-acetate buffer containing 50 mM sodium tartrate (pH 5.0) for 30 min at 37°C. TRAP-positive multinuclear cells were counted as osteoclast.

### Pit formation assay

To obtain osteoclasts, the osteoclast precursor cells were plated in calcium phosphate-coated plates (Bone Resorption Assay Plate 24) and stimulated with M-CSF and RANKL for 3 days as described above. The cells were then treated with poly(P) and further incubated for 4 days. The bright field images of five randomly selected fields per well were obtained and the pit area was measured using Image J.
